# *Lawsonia Inermis* Markedly Improves Cognitive Functions in Animal Models and Modulate Oxidative Stress Markers in the Brain

**DOI:** 10.3390/medicina55050192

**Published:** 2019-05-22

**Authors:** Numra Tariq Mir, Uzma Saleem, Fareeha Anwar, Bashir Ahmad, Izhar Ullah, Sundas Hira, Tariq Ismail, Tahir Ali, Muhammad Ayaz

**Affiliations:** 1 Riphah Institute of Pharmaceutical Sciences, Lahore Campus, Lahore 54000, Pakistan; numratariqmir@gmail.com (N.T.M.); bashir.ahmad@riphah.edu.pk (B.A.); sundas.hira@riphah.edu.pk (S.H.); tahir.ali@riphah.edu.pk (T.A.); 2 Faculty of Pharmaceutical Sciences, College of Pharmacy, Government College University, Faisalabad 38000, Pakistan; uzma95@gmail.com; 3 Department of Pharmacy, Faculty of Medical and Health Sciences, University of Poonch, Rawalakot 12420, Pakistan; izhar_pharma@yahoo.com; 4 Department of Pharmacy, Commission on Science and Technology for Sustainable Development in the South (COMSAT), Institute of Information Technology, Abbottabad 22060, Pakistan; tariqismail@ciit.net.pk; 5 Department of Pharmacy, University of Malakand, Khyber Pakhtunkhwa 18800, Pakistan

**Keywords:** *Lawsonia inermis*, nootropic, Alzheimer’s disease, SOD, CAT, oxidative stress, transfer latency, step down latency

## Abstract

*Background and Objective:* Medicinal plants represent an important source of alternative medicine for the management of various diseases. The present study was undertaken to assess the potential of *Lawsonia inermis* ethanol (Li.Et) and chloroform (Li.Chf) extracts as memory-enhancing agents in experimental animals. *Materials and Methods:* Li.Et and Li.Chf were phytochemically characterized via gas chromatography-mass spectroscopy (GC-MS). Samples were tested for nootropic potentials at doses of 25, 50, 100, 200 mg/kg (per oral in experimental animals (p.o.)). Swiss albino mice of either sex (*n* = 210) were divided into 21 × 10 groups for each animal model. Memory-enhancing potentials of the samples were assessed using two methods including “without inducing amnesia” and “induction of amnesia” by administration of diazepam (1 mg/kg, intraperitoneally. Piracetam at 400 mg/kg (i.p.) was used as positive control. Cognitive behavioral models including elevated plus maze (EPM) and the passive shock avoidance (PSA) paradigm were used. Biochemical markers of oxidative stress such as glutathione (GSH), catalase (CAT), superoxide dismutase (SOD) levels were analyzed in the brain tissue of treated mice. *Results:* In 2,2-diphenyl-1-picrylhydrazyl (DPPH) free radicals scavenging assay, Li.Et and Li.Chf exhibited 70.98 ± 1.56 and 66.99 ± 1.76% inhibitions respectively at 1.28 mg/mL concentration. GCMS results revealed the presence of important phytochemicals. Both samples (Li.Et and Li.Chf) at 25 mg/kg (p.o.) dose significantly (*p* < 0.05) improved learning and memory as indicated by decline in transfer latency and increase in step down latency in EPM and PSA models respectively. Li.Et and Li.Chf at 25 mg/kg (p.o.) showed considerable increase in GSH (2.75 ± 0.018 ***), SOD (2.61 ± 0.059 ***) and CAT (2.71 ± 0.049 ***) levels as compared to positive and negative control groups. *Conclusions:* This study provides the preliminary clue that *L. inermis* may be a potential source of memory-enhancing and anti-oxidant compounds and thus warrant further studies.

## 1. Introduction

Neurodegenerative disorders are associated with progressive decline in cognitive functions, behavioral turbulence and deterioration in routine life activates [1]. Cognitive enhancers are agents used to revitalize the brain capability to cope with memory issues. Until now, only five drugs including donepezil, rivastigmine, memantine, tacrine and galanthamine are clinically approved for the symptomatic relief of the degenerative diseases like Alzheimer’s disease (AD) [2]. Among the pathological aspects of AD is the deposition beta-amyloid (Aβ) plaques, Aβ-mediated excessive generation of reactive oxygen species (ROS), decline in essential neurotransmitter acetylcholine (ACh), hypperphosphorylated tau proteins and glutamatergic abnormalities [3,4]. The ROS species had capability to oxidize the bio-chemicals and destroy the lipids, DNA, RNA and causes degenerative changes in the body leading to cancer, cirrhosis, and brain disorders [5,6]. The immune system anti-oxidants normally scavenge these ROS, however due to excessive liberation of these ROS, or decline of immune system scavenging capacity, exogenous antioxidant therapy is necessary [7]. Anti-oxidants are responsible for nullifying the actions of free radicals and have the ability to upgrade various diseased states like neurodegenerative disorders etc [8]. The sufficient dose of anti-oxidants were reported to enhance memory [9,10]. However, currently available antioxidants are associated with bioavailability, metabolism issues and low permeability especially across the blood brain barrier (BBB) [11]. Thus there is dire need for the development of more effective antioxidants especially from natural sources.

Stress plays a vital role in provoking various disorders like neurodegenerative disorders, cardiovascular disorders and aging. As in response to stress brain recruits the various neuronal pathways that leads the activation of stress hormone and production of cortisol [12,13]. Chronic release of stress hormones causes spatial and contextual memory deficits [14]. Neurodegenerative disorders and stress are co-related with each other. Stress can result in the malfunctioning of hippocampus that is responsible in fractionation of memory [15].

Different models have been used to evaluate the memory enhancing agents such as rectangular and Y maze apparatus, open field test, Morris water maze (MWM), elevated plus maze (EPM), and passive avoidance shock (PAS) paradigm [16]. The most useful commonly used method is of EPM as it is most convenient, cheap and less time-consuming [17]. The PAS model is another useful tool for the accurate evaluation of learning and memory enhancing capacity of rodents [18]. Each model is based on particular mechanism to probe into a specific type of memory; for example, for spatial learning and memory the MWM test is usually preferred [19].

Medicinal plants have been playing a vital role in healthcare systems owing to their wide pharmacological potentials, low costs and higher safety margins [20,21,22,23,24,25]. *Lawsonia inermis* (*L. inermis*) (henna) grows well in hot and dry climates across the Arabian Peninsula, North and East Africa, South Asia, the southern areas of the Middle East [26]. It possess strong antibacterial [27], antioxidant [28], hepatoprotective [29], antiviral [30], anti-inflammatory [31], and anti-parasitic potentials. In traditional medicine it is used as a memory-enhancing agent [32]. Various chemical constituents have been derived from the leaves of *L. inermis* which might be responsible for its scavenging and other pharmacological potentials [33]. The current study was aimed to appraise the memory-enhancing potentials of *L. inermis* ethanol and chloroform extracts using EPM and PAS models. The effect of the tested samples on the oxidative stress biochemical markers, glutathione (GSH), superoxide dismutase (SOD) and catalase (CAT) were analyzed in the brain tissues of the animals. 

## 2. Materials and Methods

### 2.1. Drugs and Chemicals

Diazepam (Valium) and Piracetam (Nootropil) injection were purchased from local pharmacy. Ethanol, sulphuric acid, carboxymethyl cellulose, chloroform, iodine, sodium hydroxide, folin-ciocalteu’s reagent, potassium acetate, methanol, formalin, ferric chloride, n-hexane, sodium carbonate, HCL, ninhydrin reagent, ferric cyanide, and 2,2-diphenyl-1-picrylhydrazyl (DPPH) were purchased from Fisher chemicals (Loughborough, UK), Ascorbic Acid, Gallic acid, Rutin and Trichloroacetic acid were obtained from Sigma Aldrich chemicals (Darmstadt, Germany).

### 2.2. Plant Collection

Plant material was purchased from local market in Islamabad Pakistan. Plant identification and authentication was done by Hassan Mushtaq, a plant taxonomist in the herbarium of Riphah International University. The plant sample was deposited at the same herbarium with voucher number PHM-COL-0002 for future records.

#### Extract Preparation

Leaves of *L. inermis* were shade dried and ground to coarse powder using a cutter mill. Powdered plant material (200 g) was macerated in 500 mL of 80% ethanol with occasional stirring for 72 h. The extract was filtered through gauze and by suction filter (G 180-US, Jonesboro, AR, USA) using Whatmann filter paper (No.1, Tisch Scientific, North Bend, OH, USA). After filtration, ethanol was removed by using a rotary evaporator (Laborota 4000, Heidolph, Schwabach, Germany) under reduced pressure. A dark brown sticky residue after extraction was lyophilized by using lyophilizer. Dried dark brown powder residue weighing 10 g was collected with a percentage yield of 5%. The extract was stored in a refrigerator for further use. Chloroform extract was prepared by the same method. A light brown powder residue weighing 5 g was obtained with a percentage yield of 2.5%. Dried powder of each extract was stored in air-tight containers for future use [34].

### 2.3. Gas Chromatography-Mass Spectroscopy (GC-MS) Analysis

Different phytochemical constituents were identified using the gas chromatography-mass spectroscopy (GC-MS) analysis [35]. GCMS-QP2010 ultra (Shimadzu, Deutschland, Germany) was used as gas chromatogram mass spectrometer. GC-MS was fused with silica column of about 25 µm with the thickness of 0.25 µm. An electron multiplier detector was used. Helium column flow was 15 mL/min. oven temperature was set at 220 °C. One microliter plant sample in n-hexane was injected and initially analyzed at 60 °C for the first 5 min then increased to 5 °C/min till 220 °C. Helium was used as a gas. Different phytochemical constituents were analyzed with their retention times.

### 2.4. Experimental Animals

Swiss albino mice of either sex weighing 27–35 g were used in the study. Animals were obtained from the animal house of University of Veterinary and Animal Sciences Lahore (UVAS). The mice were kept in the animal house of Riphah International University Lahore one week prior to the study for acclimatization. The mice were placed in plastic cages at temperature 25 ± 2 °C and 45 ± 5%. The mice had free access to food and water. The care and handling of animals was done following international guidelines [36]. This study was carried out on animals after approval from the research ethical committee of Riphah International University with an authorized number of REC/RIPS-LHR/2017/002 (approved on 15 March 2018) ruled under the regulation of the National Institutes of Health guide for the care and use of Laboratory animals (NIH Publications No. 8023, revised 1978) and following ARRIVE guidelines (Supplementary File S1).

#### 2.4.1. Acute Toxicity

Keeping in mind the toxicological aspects of the plant, an acute toxicity study was conducted according to (OCED) Organization of Economic Corporation and Development 425 guidelines. Experimental animals were treated with the challenging dose of 2000 mg/kg of both extracts of *L. inermis* through the oral route. After the administration of the dose, mortality was observed in the first initial 24 hours till the 14th day [37].

#### 2.4.2. Study Design

Mice were divided into 21 groups. Each group comprised 10 mice. The experimental protocol was divided into two models.

#### 2.4.3. Estimation of Memory-Enhancing Activity without Induction of Amnesia

**Group I** was designated as control group and received 5% carboxymethy cellulose (CMC). 

**Group II** was assigned as positive control and received standard drug (piracetam 400 mg/kg, intraperitoneal (i.p.).

**Groups III–VI** were treated with *L. inermis* ethanol (Li.Et) at dose level of 25, 50, 100, 200 mg/kg respectively for 15 consecutive days orally.

**Groups VII–X** were treated with chloroform (Li.Chf) at dose level of 25, 50, 100, 200 mg/kg respectively for 15 consecutive days orally.

#### 2.4.4. Estimation of Memory-Enhancing Activity in Diazepam-Induced Amnesia Model

Amnesia was induced in the disease control group and treatment groups by administering diazepam 1 mg/kg (i.p.) on alternate days during the whole study. Study design was as follows:

**Group I,** served as control group which received 5% CMC.

**Group II** was assigned as positive control which was given a standard drug (piracetam 400 mg/kg, i.p.).

**Group III** disease control, receiving diazepam (1 mg/kg, i.p.).

**Groups IV–VII** were treated with Li.Et at dose level of 25, 50, 100, 200 mg/kg respectively for 15 consecutive days orally.

**Groups VIII–XI** treated with Li.Chf at a dose level of 25, 50, 100, 200 mg/kg respectively for 15 consecutive days orally.

### 2.5. Models for Behavioral Studies

#### 2.5.1. Elevated plus Maze (EPM) Task

The elevated plus maze was used to evaluate the memory enhancing capacity of the test samples. EPM is composed of two open (16 × 5 cm) and two closed arms (16 × 5 ×12 cm). EPM arms are 5 × 5 cm apart from the central platform. The apparatus is elevated to the height of 30 cm above the floor [38]. For modifications, open arms were painted white and closed arms were painted black.

#### 2.5.2. Training and Test Sessions

Training was done for nine consecutive days and the test session was conducted after 90 min of the last dose on 15th day. The animal was placed on the open arm. The time taken by the animal to enter the closed arm (TL) was observed. A cut-off time period of 90 s was chosen for the animals i.e., if animal did not enter the closed arm with in 90 s, the animal was replaced from the study. Memory-retaining capability of each mouse was recorded 24 h after the training session on the second day (16th day) [39].

### 2.6. Passive Shock Avoidance Paradigm

The PSA tool is composed of a square shaped container (27 × 27 × 27 cm) enclosed in triple wooden walls, along with a base of stainless steel having rods. The rods were 3 mm long and 6 mm separated from each other. The base of wood was (10 × 7 × 1.7 cm) wide. A 15 W bulb was used to illuminate the wooden box during the experiment. A current of 20 V AC was supplied to the grid floor for providing the shock to the animals [40].

#### Training and Test Sessions

Training was done for nine consecutive days and a test session was given to each mouse after 90 min of the last dose on the 15th day. Each mouse was placed on the wooden base at the center of grid. When the mouse stepped down on the grid floor with all its paws, a current was supplied to the grid floor for 15 s. Step down latency was recorded i.e., the time taken by the mouse to come down from the wooden base to the grid floor [41].

### 2.7. Assessment of Biological Indicators of Oxidative Stress

#### 2.7.1. Brain Tissue Preparation

After the experimental procedures, animals were sacrificed by giving mild anesthesia. The brain was removed and put in ice-cold normal saline solution to remove the debris. A 1/10 (*w/v*) tissue homogenate was prepared in (0.1 M phosphate buffer, pH 7.0) Tissue homogenates were centrifuged (T6-3A-US, Galavano Scientific, Lahore, Pakistan) at 600× *g* for 10 min at 4 °C. Supernatants were collected and used for biochemical estimation [42].

#### 2.7.2. Estimation of Glutathione

Tissue homogenates (1 mL) were mixed with 0.02 mol/L ethylene diamine tetra acetic acid (EDTA) and tubes were placed in ice bath for 10 min to precipitate the tissue proteins. Subsequently, 2 mL distilled water and 0.5 mL of tri-chloroacetic acid (50%) were added in the test tube and again placed in an ice bath for 10–15 min and centrifuged at 3000–3500 rpm/min. Supernatant was collected and absorbance was recorded at 412 nm. A blank was prepared by the same method without the addition of analyte [43].

$$\mathrm{GSH}\;\mathrm{level}=\frac{Y\_0.00314}{0.0314}\times \frac{\mathrm{DF}}{\mathrm{BT}}\times \mathrm{VU}$$

where, Y is Absorbance of tissue homogenate, DF is dilution factor (1), BT is brain tissue homogenate (1 mL), VU is aliquot Volume (1 mL) [44].

#### 2.7.3. Estimation of Superoxide Dismutase (SOD)

Sodium phosphate buffer (0.025 mol/L, pH 8.3) 1.25 mL, tissue homogenate 200 µL, phenazine methosulphate (186 µmol/L) 1.25 mL and nitro blue tetrazolium chloride (NBT) (300 µL) were taken in test tube. Then, (780 µmol/L), 200 µL reduced nicotinamide adenine dinucleotide was added to the mixture to start the reaction. The reaction mixture was incubated at 30 °C for 90 s. Furthermore, 1 mL glacial acetic acid was added in the reaction mixture to stop the reaction. The reaction mixture was shaken with 4 mL of *n*-butanol and incubated for 10 min at room temperature. The n-butanol layer was removed and intensity of chromogen was measured at wavelength 560 nm. A blank was prepared by the same method without adding tissue homogenate [45].

#### 2.7.4. Estimation of Catalase (CAT)

Tissue homogenate, 100 µL, phosphate buffer pH 7 and 1.9 mL freshly prepared H_2_O_2_ were transferred in cuvette. Blank and standard were prepared similarly without addition of tissue homogenate respectively. Absorption of samples was measured against a blank at 240 nm [43]. Following formula was used to calculate CAT activity:

$$\mathrm{CAT}\;\mathrm{activity}=\frac{\mathrm{OD}}{\mathrm{Ex}\;\mathrm{Vol}.\;\mathrm{of}\;\mathrm{sample}\;\left(\mathrm{mL}\right)\;}\times \mathrm{mg}\;\mathrm{of}\;\mathrm{protein}$$

where OD is change in absorbance/min, Ex is extinction coefficient of hydrogen peroxide (0.071 mmol/cm) [44].

### 2.8. In Vitro 2,2-diphenyl-1-picrylhydrazyl (DPPH) Free Radical Scavenging Assay

Different concentrations (0.02–1.28 mg/mL) of Li.Et and Li.Chf were used to study the anti-oxidant potentials. DPPH was prepared in methanol. DPPH (3 mL) and 1 mL of each concentration were poured into their respective test tubes. The mixture was incubated at ambient temperature for half an hour. Blank was prepared by the same method without the addition of analyte. Absorbance was recorded at wavelength of 715 nm. Lesser absorbance indicates more antioxidant activity [46]. Rutin was used as positive control.

$$\%\;\mathrm{Scavenging}\;\mathrm{effect}=\frac{A\;\mathrm{blank}\;-\;A\;\mathrm{sample}\;}{A\;\mathrm{blank}}\times 100$$

### 2.9. Statistical Analysis

Data are represented as mean ± SEM (*n* = 10). One-way analysis of variance (ANOVA) followed by Dunnett’s *t* test was applied on data by using graph pad prism version 5.0 Data were considered statistically highly significant at *p* < 0.001, moderately significant at *p* < 0.01, and significant at *p* < 0.05 [47].

## 3. Results

### 3.1. GC-MS Analysis of *L. Inermis*

**Figure 1:**
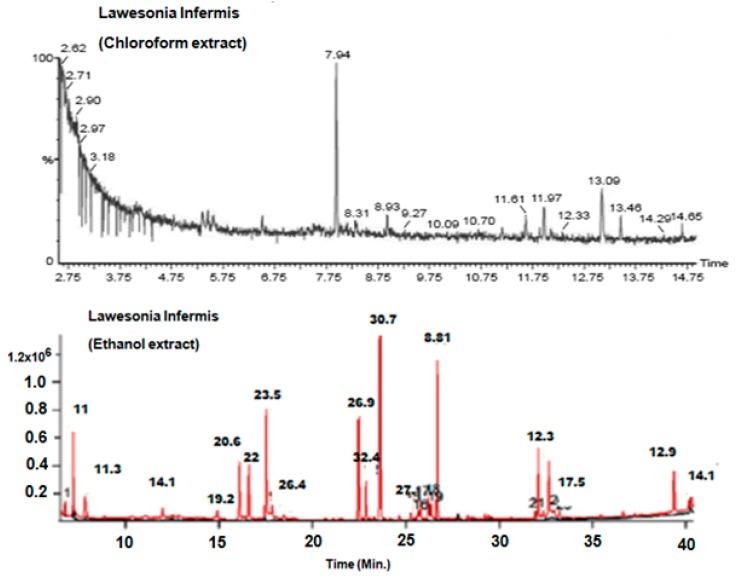
Gas chromatography-mass spectroscopy (GC-MS) chromatograms of Li.Chf. and Li.Et. GC-MS, gas chromatography-mass spectroscopy; Li.Et, *Lawsonia inermis* ethanol; Li.Chf, chloroform.

The GC-MS study of Li.Et and Li.Chf exhibited several phytochemicals in Li.Chf and Li.Et (Figure 1, Table 1 and Table 2). Compounds were identified by comparing their retention times, fragmentation pattern of mass spectra’s with NIST and Wiley libraries and published literature [48]. In the phytochemical analysis of Li.Chf, 12 compounds were identified. Among these, phytol, pseudoephedrine, aspidofractinine-3-methanol, phenol, 2,6-bis(1,1-dimethylethyl)-4-methyl-, methylcarbamate were in the highest concentrations. The highest peak of phenol was 2,6-bis (1,1-dimethylethyl)-4-methyl-, a methyl carbamate compound that is phenolic in nature and may be implicated in the anti-oxidant and nootropic potentials of the extract. Phytol was the highest concentration compound in both extracts. In the phytochemical analysis of Li.Et, 16 compounds were identified. Among these, 3,7,11,15-tetramethyl-2-hexadecen-1, E-2-tetradecen-1-ol, 2-tridecen-1-ol, phytol, 1-eicosanol,Z,Z-2, 5-pentadecadien-1-ol, 3-hexadecyloxy-carbonyl-5- (2-hydroxyethyl)-4 -methyl imidazolium ion and squalene were the most abundant compounds.

### 3.2. Acute Toxicity

Results of acute toxicity studies showed that Li.Et and Li.Chf were safe up to 2000 mg/Kg. No mortality was observed within 24 hrs immediately or up to 14 days of observation. No symptoms like loss of body weight, salivation, paralysis, tremors were observed. The Li.Et and Li.Chf have LD_50_ of greater than 2000 mg/Kg.

### 3.3. Effect of Lawsonia Inermis Ethanol (Li.Et) and Lawsonia Inermis Chloroform (Li.Chf) on Transfer Latency Using EPM Paradigm

**Figure 2:**
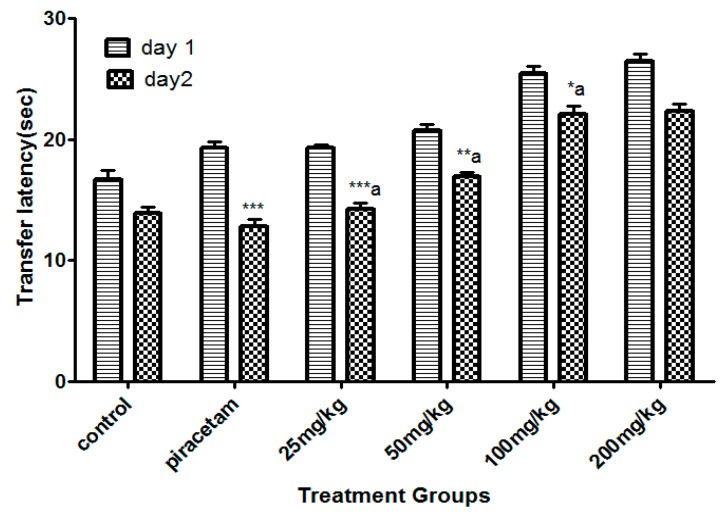
Effect of Li.Et therapy on transfer latency (TL) of mice in the elevated plus maze (EPM) model. Piracetam (400 mg/kg, i.p.) was used as a positive control. Results are presented as mean ± standard error of the mean (SEM) (*n* = 10). One-way analysis of variance (ANOVA) followed by Dunnett’s *t* test was used for statistical analysis of data. *** indicates *p* < 0.001, ** indicates *p* < 0.01, and * indicates *p* < 0.05 in comparison with control. a: indicates *p* < 0.001 in comparison with Piracetam.

**Figure 3:**
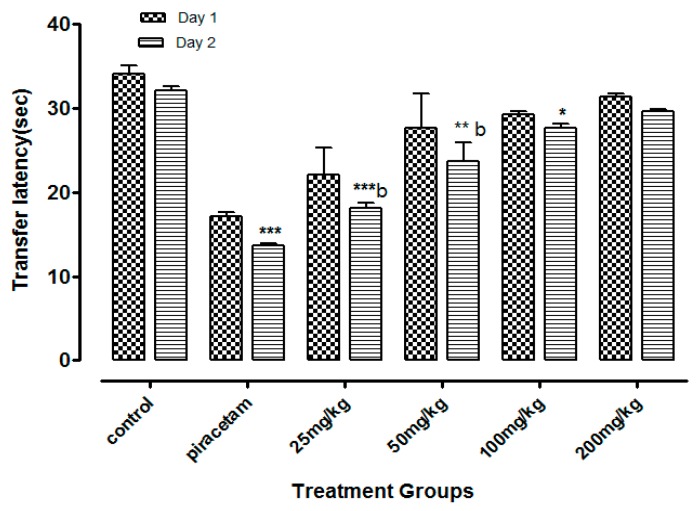
Effect of Li.Chf on transfer latency (TL) of mice using elevated plus maze model. Results are presented as Mean ± SEM (*n* = 10). One-way ANOVA followed by Dunnett’s *t* test was used for statistical analysis of data. *** indicates *p* < 0.001, ** indicates *p* < 0.01 and * indicates *p* < 0.05 in comparison with control. b: indicates *p* < 0.001 in comparison with Piracetam.

The least dose of Li.Et at 25 mg/kg (p.o.) displayed a considerable decline (*p* < 0.001) in the transfer latency (TL) (14. 28 ± 0.65 s) of animals in comparison with the piracetam group with TL of 13.56 ± 0.88 s. Li.Et exhibited a dose-dependent decline in TL at 50, 100 and 200 mg/kg as shown in (Figure 2). Further, Li.Chf at 25 mg/kg (p.o.) exhibited TL of 18.14 ± 0.68 s which was significantly different (*p* < 0.001) in comparison with piracetam with TL of 13.68 ± 0.34 s (Figure 3).

**Figure 4:**
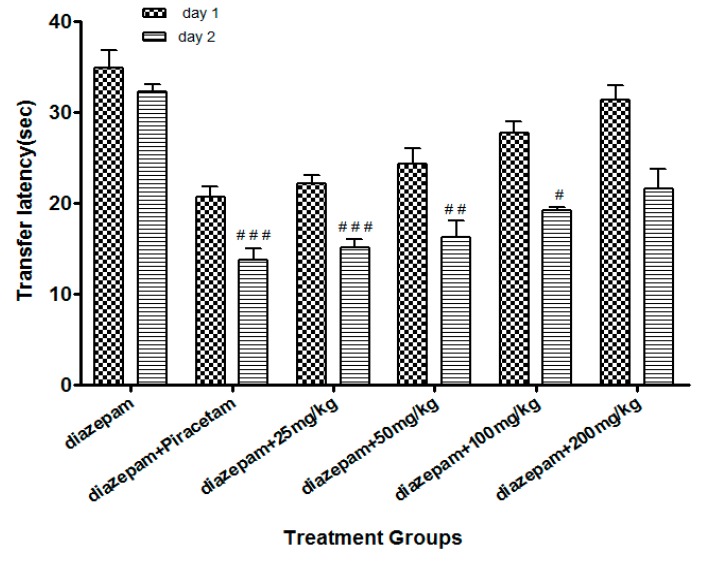
Effect of Li.Et on transfer latency (TL) in mice using the EPM model. Data is presented as Mean ± SEM (*n* = 10). One-way ANOVA followed by Dunnett’s *t* test was used for statistical analysis of data. ### indicates *p* < 0.001, ## indicates *p* < 0.01 and # indicates *p* < 0.05 in comparison with diazepam.

**Figure 5:**
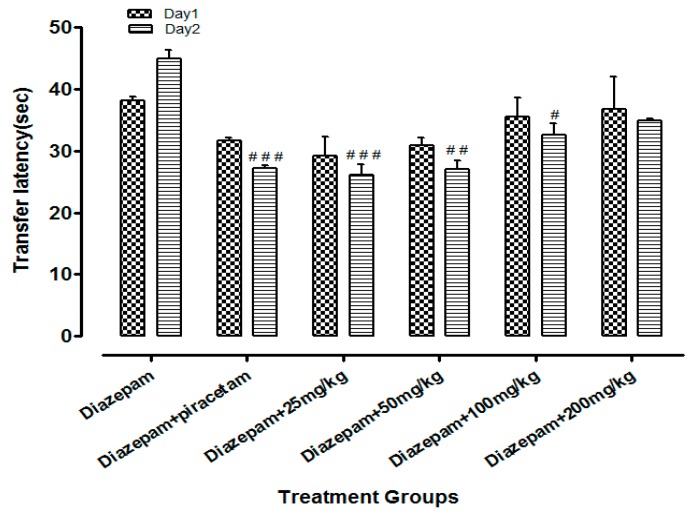
Effect of Li.Chf on transfer latency (TL) of mice using EPM model. Results are presented as Mean ± SEM (*n* = 10). One-way ANOVA followed by Dunnett’s *t* test was used for statistical analysis of data. ### indicates *p* < 0.001 in comparison with diazepam. ## indicates *p* < 0.01 in comparison with diazepam. # indicates *p* < 0.05 in comparison with diazepam.

Li.Et (25mg/kg) in combination with diazepam (1 mg/kg) exhibited a steady decline in TL (15.06 ± 1.24 s in comparison to diazepam group only, with TL of 32.02 ± 1.20 s. Diazepam (1 mg/kg in combination with piracetam at 400 mg/kg was most effective in reducing the TL (13.77 ± 1.53 s) of rodents. Among the other group, a concentration-dependent effect on TL was observed (Figure 4). Likewise, the TL was 44.90 ± 1.82 s in diazepam treated group, which was significantly reduced by Li.Chf (25 mg/kg) in combination with 1 mg/kg diazepam to 26.08 ± 2.33 s (Figure 5). Piracetam at 400 mg/kg in combination with 1 mg/kg diazepam reduced TL to 27.11 ± 0.58 s in comparison to the control group. All other group animals at 50, 100 and 200 mg/kg exhibited a dose-dependent decline in TL.

### 3.4. Effect of Li.Et and Li.Chf on Step-Down Latency Using Passive Avoidance Paradigm

**Figure 6:**
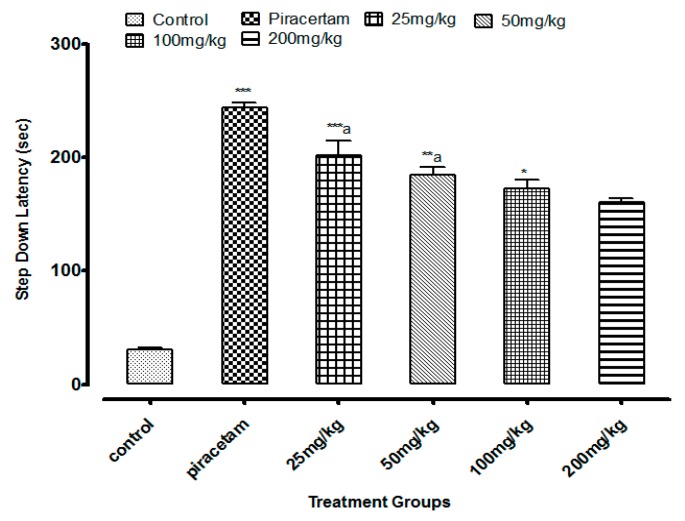
Effect of Li.Et on step down latency (SDL) in mice using passive avoidance model. Piracetam (400 mg/kg, i.p.) was used as a positive control**.** Results are presented as Mean ± SEM (*n* = 10). One-way ANOVA followed by Dunnett’s *t* test was used for statistical analysis of data. *** indicates *p* < 0.001, ** indicates *p* < 0.01 and * indicates *p* < 0.05 in comparison with control. a, indicates *p* < 0.001 in comparison with Piracetam.

**Figure 7:**
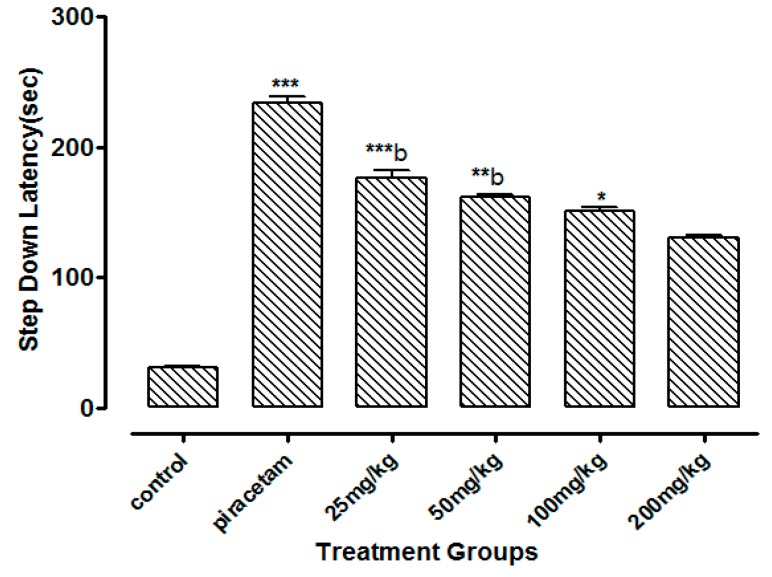
Effect of Li.Chf on step down latency (SDL) in mice using passive avoidance model. Piracetam (400 mg/kg, i.p.) was used as positive control. Results are presented as Mean ± SEM (*n* = 10). One-way ANOVA followed by Dunnett’s *t* test was used for statistical analysis of data. *** indicates *p* < 0.001, ** indicates *p* < 0.01 * indicates *p* < 0.05 in comparison with control. b, indicates *p* < 0.001 in comparison with Piracetam.

**Figure 8:**
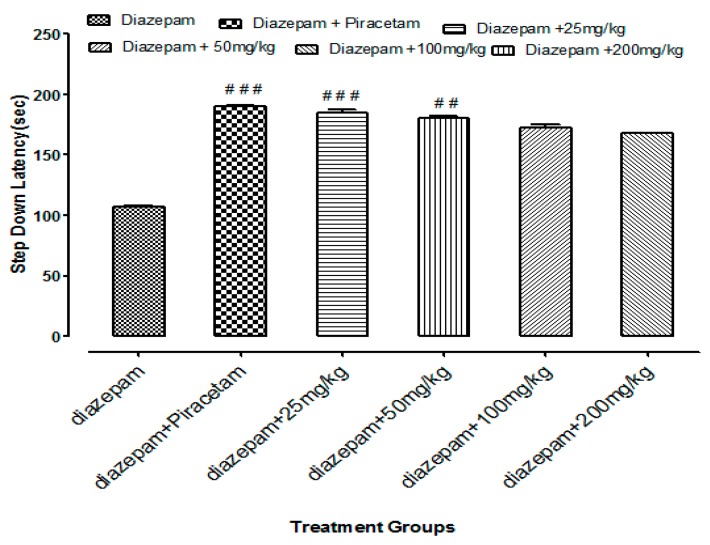
Effects of Li.Et on step down latency (SDL) of mice treated with diazepam using passive avoidance model. Results are presented as Mean ± SEM (*n* = 10). One-way ANOVA followed by Dunnett’s *t* test was used for statistical analysis of data. ### indicates *p* < 0.001 in comparison with diazepam.## indicates *p* < 0.01 in comparison with diazepam. # indicates *p* < 0.05 in comparison with diazepam.

Li.Et at 25 mg/kg dose exhibited a highly significant increase of 201.62 ± 1.56 s (*p* < 0.001) in step down latency (SDL) of rodents as compared to control group animals with SDL of 30.78 ± 0.58 s. However, positive control piracetam exhibited SDL of 244.08 ± 2.33 s. Other concentrations of Li.Et showed dose dependent but less significant increase in SDL of animals (Figure 6). Further, control group, piracetam (400 mg/kg), Li.Chf (25 mg/kg), Li.Chf (50 mg/kg), Li.Chf (100 mg/kg) and Li.Chf (200 mg/kg) groups exhibited 30.78 ± 0.56, 233.51 ± 2.77, 176.46 ± 0.88, 162.03 ± 0.33, 151.38 ± 0.55, 130.31 ± 1.05 s SDL respectively (Figure 7). Li.Et at 25mg/kg exhibited a steady improvement in SDL (184.57 ± 2.12 s) when administered after administration of 1 mg/kg diazepam. The SDL in diazepam-treated animals was 106.84 ± 0.36 s whereas the piraacetam group exhibited 189.71 ± 2.22 s SDL when combined with diazepam (Figure 8).

### 3.5. Assessment of Biological Indicators of Oxidative Stress 

#### 3.5.1. Estimation of Glutathione (GSH)

Li.Et at dose of 25 mg/kg, exhibited a highly significant (*p* < 0.001) increase in GSH in comparison with diazepam. However, the groups in which disease was induced by diazepam (1mg/Kg) along with the administration of dose (25 mg/kg, p.o.) exhibited a highly significant increase in GSH levels as compared to the disease group. The dose (50 mg/kg, p.o.) along with the administration of diazepam showed a moderately significant (*p* < 0.01) increase in GSH levels as compared to diazepam (Table 3).

#### 3.5.2. Estimation of SOD

The Li.Et and Li.Chf lowest dose (25 mg/kg, p.o.), showed a highly significant (*p* < 0.001) increase in SOD levels in comparison with diazepam. The disease-induced group along with the administration of dose 25 mg/kg, p.o.) displayed highly significant (*p* < 0.001) increase in SOD levels as compared to diazepam (Table 3).

#### 3.5.3. Estimation of CAT

Li.Et and Li.Chf at 25 mg/kg, p.o. dose showed a highly significant (*p* < 0.001) increase in CAT levels in comparison with diazepam (Table 3).

### 3.6. DPPH Free Radicals Scavenging Activity 

**Figure 9:**
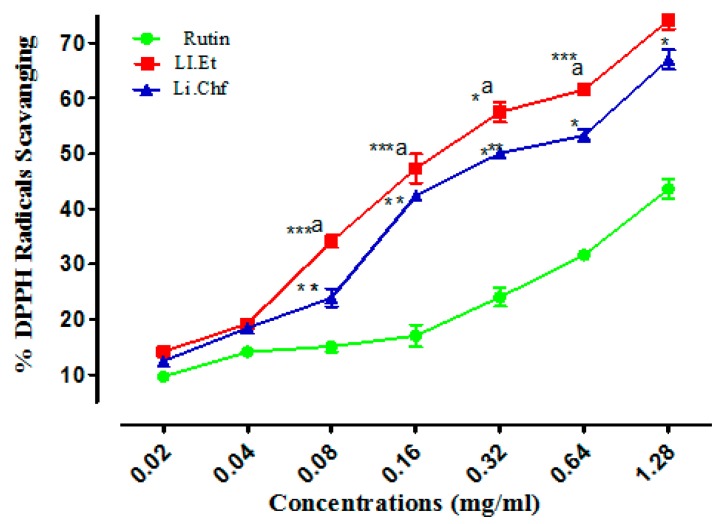
Results of DPPH radical scavenging activity (%) of Li.Et and Li.Chf. Superscript. *** represent *p* < 0.001, ** represent *p* < 0.01 and * represent *p* < 0.05 in comparison with standared agent rutin Superscript a represent P < 0.01 in comparison of Li.Et with Li.Chf. Values are expressed as Mean ± SEM (*n* = 5).

In DPPH radicals scavenging assay, Li.Et showed 74.1 ± 1.56%, 61.56 ± 0.034% inhibitions at the highest concentrations of 1.28 and 0.64 mg/mL, respectively. All other concentrations of Li.Et inhibited free radicals in a concentration-dependent manner (Figure 9). Likewise, Li.Chf at concentrations of 1.28 and 0.64 mg/mL exhibited 66.99 ± 1.76% and 53.28 ± 1.13% inhibition of DDPH radicals, respectively. Rutin displayed 43.57 ± 1.77% inhibition at 1.28 mg/mL against DPPH radicals. Several phenolic compounds have been reported from this plant, which might act as free-radical scavengers. The flavonoids from dietary sources exhibit anti-oxidant properties [49]. The anti-oxidant activity of *L. inermis* might be due to the presence of flavonoids. A low dose (25 mg/kg, p.o.) of both extracts displayed a highly significant (*p* < 0.001) increase in memory. Further studies are required to study the exact mechanism of action.

## 4. Discussion

Loss of memory and cognitive dysfunctions lead to progressive neurodegenerative disorders like AD [50]. Neurodegenerative symptoms arise due to improper neurogenesis in the hippocampus [51]. Aging is also a cause of memory loss and its burden is increasing day by day in the public due to stressful lifestyles [52]. Several behavioral tasks including EPM, PAT are among the most extensively used tools to assess the cognitive performance of rodents. EPM comprising covered and open arms was utilized for the estimation of stress and nootropic effect in animals. Mice escaped from the exposed arm towards the covered one. The interval of time that the animal took to escape the exposed arm towards the covered (transfer latency) was recorded. The transfer latency on the 2nd day significantly declined as compared to the 1st day, indicating improvement in memory and reduction in stress. Transfer latency on the 2nd day was not reduced in mice treated with diazepam (1 mg/kg, i.p.) indicating impairment in memory. Its working principle includes the natural ability of rodents to find protective environments and their natural ability to reject bright, unprotected, and elevated places (shown by the open arms). Confinement to the open arms generates physiological signs of stress (increased corticosterone levels and defecation), whereas exposure to memory-enhancing drugs, such as pirecetam increases exploration of these arms [53]. Both of our test samples at dose of 25 mg/kg, p.o. significantly (*p* < 0.001) enhanced the memory as compared to control and standard groups on the 15th day. Decrease in TL indicated enhancement in memory. Li.Et and Li.Chf displayed dose independent nootropic effects as shown in Figure 2, Figure 3, Figure 4 and Figure 5.

It is well documented that diazepam cause impairment in retrieval of memory in mice and such amnesia is associated with a significant increase in oxidative stress. Continuous stress can lead to amnesia that resulted in memory loss [54]. Those groups treated with diazepam along with administration of oral doses of test samples (25, 50, 100, 200 mg/kg) indicated reversal of amnesia induced by diazepam (Figure 2, Figure 3, Figure 4 and Figure 5). The least dose (25 mg/kg, p.o.) produced a highly significant decrease in TL as compared to diazepam alone. The effect on low dose might be due to the attenuation of auto receptors of acetylcholine on presynaptic neurons that inhibited the negative feedback of neurotransmitter release, while at a high dose it might potentiate the post-synaptic receptor stimulation that destroyed the acetylcholine rapidly and resultantly decrease the nootropic potential of *L. inermis* [55].

Passive avoidance is mostly used to estimate the cognitive effects of test samples on rodents. It is a simple and flexible method for evaluation of nootropic potential in the field of neuropharmacology and other fields. It can be utilized to evaluate both the diminutive along with longstanding memory. In the present study low doses of Li.Et and Li.Chf at 25 mg/kg, p.o. significantly (*p* < 0.001) enhanced the SDL as compared to control and standard. Those groups treated with diazepam along with the administration of oral doses (25, 50, 100, 200 mg/kg) indicated reversal of amnesia induced by diazepam. The lowest dose (25 mg/kg, p.o.) displayed a highly significant (*p* < 0.001) increase in SDL as compared to diazepam alone.

Factors responsible for inducing the neuronal changes seem to be free radicals. Various enzymes are responsible for radical scavenging of free radicals; these include CAT, GSH and SOD. Impairment in the proper functioning of these enzymes gives rise to the augmentation of free radicals [56]. Previous studies have revealed that stress and free radicals are responsible for impairing memory and learning. Stress can lead to a disturbance in levels of certain neurotransmitters [57]. Glutathione plays a significant role in the detoxification of free radicals thus reducing stress and enhancing cellular functions. Glutathione levels are reduced in diseased conditions leading to oxidative stress [58]. Results demonstrated that the GSH level was increased significantly (*p* < 0.001) in group treated with Li.Et and Li.Chf (25 mg/kg, p.o.) as compared to diazepam. An increase in GSH level indicated a reduction in stress and an enhancement in memory (Table 3).

SOD plays a vital role in shielding the living cells against the toxicity generated by the free radicals due to their capability to scavenge O^−2^ [59]. SOD levels were determined from brain tissue homogenates of mice. The least dose (25 mg/kg, p.o.) displayed highly significant increase in SOD level as compared to diazepam. CAT activity was estimated and linked with the nootropic activity of *L. inermis* in mice. The lowest dose (25 mg/kg, p.o.) exhibited more pronounced increase in CAT activity as compared to diazepam. In the case of oxidative stress the levels of CAT, SOD and GSH are reduced which can lead to death of neurons leading to memory impairment. *L. inermis* (25 mg/kg, p.o.) of both extracts enhanced the levels of these enzymes. Thus, *L. inermis* can be used as a nootropic agent.

The role of free radicals in the etiology of neurodegenerative disorders has been understood for a long time and the role of antioxidants in the management of neurodegenerative disorders has been reported time and again in the last few decades. A lot of natural and synthetic compounds have been showing promising results in the scavenging of free radicals but almost all the compounds have been associated with certain shortcomings [60]. One of the most prominent limitations of the antioxidant compounds is their highly polar nature due to the presence of polar functional groups which confine the flow of the antioxidants either to the blood stream and do not allow their entry to the target point that is the central nervous system. Among the natural compounds, the flavonoids have been showing promising in vitro results against the free radicals but fail to decrease the oxidative stress in the central nervous system (CNS) due to the presence of phenolic groups in the flavonoids which make them highly polar and which even decreases their bioavailability [61]. Secondly, there are numerous antioxidant compounds which have the ability to capture the free electrons from the free radicals, but the problem is that these compound do not have the capacity to remain stable after gaining the free electrons from free radicals [11]. This may lead to the generation of other metabolites which may be more hazardous than the oxidative stress. Researchers are striving for the development of various drug-delivery systems and blood brain barrier models to cope with the bioavailability and CNS delivery issues. Moreover, there is no role for antioxidants in reversing nerve damage, albeit the antioxidant compounds could be able to halt further exacerbation. Likewise, the radical scavenging antioxidants are oxidant specific and can only be effective if the specific mechanism for neurode generation involves the reactive species to which they are targeted. Most importantly, the suppression of oxidants may be deleterious for the human body, as we know that the reactive species have an important role in physiological signalling within the body [62,63]. In the current study, our test samples considerably augmented the antioxidant activity of indigenous antioxidant enzymes in the brain homogenates of the animals, which indicates that they effectively cross the BBB to gain access to the brain.

## 5. Conclusions

GC-MS analysis of Li.Etand Li.Chf revealed the presence of phytochemical constituents that may be responsible for the nootropic activity of *L. inermis*. It was concluded from the study that Li.Etand Li.Chf of *L. inermis* had the ability to reverse the memory loss due to free radical-induced neurodegeneration. The exact mechanism of action is not known; however, the anti-oxidant property of *L. inermis* might be responsible for the nootropic potential of the plant. The isolation of active phyto-constituents, their structure elucidation, and the exact mechanism by which extracts modulate Alzheimer’s disease are limitations of the current study.

## Figures and Tables

**Table 1:** Gas chromatography-mass spectroscopy (GC-MS) analysis and phytochemical constituents in Li.Chf.

Serial. No.	Ret. Time (min)	Name of Compound	Molecular Formula	Molecular Weight	Peak Area (%)
1	4.61	isoxazolidine, 5-hexyl-, (+)	C_9_H_19_NO	157	0.47
2	4.79	1,2,4-butanetriol, trinitrate	C_4_H_7_N_3_O_9_	241	0.47
3	4.91	N-ethyl-N’-nitroguanidine	C_3_H_8_N_4_O_2_	132	0.47
4	7.94	phenol, 2,6-bis(1,1-dimethylethyl)-4-methyl-, Methylcarbamate	C_17_H_27_NO_2_	277	2.37
5	8.31	1,3-dimethyl-3-ethyl, 2,5-Pyrrolidinedione	C_8_H_13_NO_2_	155	0.16
6	8.93	2-Piperidinone, N-[4-bromo-n-butyl]	C_9_H_16_BrNO	233	0.16
7	11.16	3-hexadecyloxycarbonyl-5-(2-hydroxyethyl)-4-methylimidazolium ion	C_24_H_45_N_2_O_3_	409	0.16
8	11.97	2-(3-Oxo-3-phenylpropyl)-3,5,6-trimethylpyrazine	C_16_H_18_N_2_O	254	0.47
9	13.09	pyridine-3-carboxamide, 4-dimethylamino-N-(2,4-difluorophenyl)	C_14_H_13_F_2_N_3_O	277	1.11
10	14.96	phytol	C_20_H_40_O	296	84.97
11	19.82	pseudoephedrine, (+)	C_10_H_15_NO	165	2.06
12	20.85	aspidofractinine-3-methanol, (2à, 3á, 5à)	C_20_H_26_N_2_O	310	7.12

GC-MS, gas chromatography-mass spectroscopy; Li.Chf, chloroform.

**Table 2:** GC-MS analysis and phytochemical constituents in Li.Et.

S. No	Ret. Time (min)	Name of Compound	Molecular Formula	Peak Area (%)
1	11	3,7,11,15-Tetramethyl-2-hexadecen-1-old	C_20_H_40_O	13.51
2	11.3	E-2-Tetradecen-1-ol	C_14_H_28_O	7.73
3	14.1	2-Tridecen-1-ol, (E)-	C_13_H_26_O	6.26
4	19.2	Phytol	C_20_H_40_O	39.1
5	20.6	1-Eicosanol	C_20_H_42_O	2.75
6	22	Z,Z-2,5-Pentadecadien-1-old	C_15_H_28_O	1.72
7	23.5	3-Hexadecyloxycarbonyl-5-(2-hydroxyethyl)-4-methylimidazolium ion	C_24_H_45_N_2_O_3_	1.42
8	26.4	Squalene	C_30_H_50_	2.42
9	26.9	6,9,12-Octadecatrienoic acid, phenylmethyl ester, (Z,Z,Z)-	C_25_H_36_O_2_	4.38
11	32.4	1b,5,5,6a-Tetramethyl-octahydro-1-oxa-cyclopropa[a]inden-6-o	C_13_H_20_O_2_	3.53
12	30.7	Benzenemethanol, 2-(2-aminopropoxy)-3-methyl-	C_11_H_17_NO_2_	2.02
13	27.1	9-Oxabicyclo[3.3.1]nonan-2-one, 6-hydroxy-	C_8_H_12_O_3_	2.14
14	8.81	Pentanal	C_5_H_10_O	4.73
15	12.3	Benzeneethanamine, 2-fluoro-á,3-dihydroxy-N-methyl-	C_9_H_12_FNO_2_	2.59
16	17.5	2-Aminononadecane	C_19_H_41_N	1.14
17	12.9	Cyclopenta[c]furo[3′,2′:4,5]furo[2,3-h][1]benzopyran-11(1H)-one, 2,3,6a,9a-tetrahydro-1,3-dihydroxy-4-methoxy-	C_17_H_14_O_7_	2.23

Li.Et, *Lawsonia inermis* ethanol.

**Table 3:** Estimation of superoxide dismutase (SOD), catalase (CAT) and glutathione (GSH) levels in Li.Et-treated mice brain tissue.

Groups	Treatment	Dose	SOD (µg/mg)	GSH (nM/mg of protein)	CAT (µg/mg)
I.	Control	5% CMC	1.34 ± 0.009	1.27 ± 0.005	1.58 ± 0.003
II.	Piracetam	400 mg/kg, p.o.	2.47 ± 0.026 *** (↑ 88.6%)	3.05 ± 0.01 *** (↑ 90%)	2.87 ± 0.016 *** (↑ 93%)
VII.	Diazepam	1 mg/kg, i.p.	0.28 ± 0.009	0.28 ± 0.008	0.19 ± 0.009
III.	Li.Et	25 mg/kg, p.o.	2.61 ± 0.059 *** (↑ 89.2%)	2.75 ± 0.018 *** (↑ 87%)	2.71 ± 0.049 *** (↑ 91%)
IV.	Li.Et	50 mg/kg, p.o.	1.13 ± 0.006 ** (↑ 75.2%)	1.67 ± 0.019 ** (↑ 83%)	1.94 ± 0.004 ** (↑ 89%)
IX.	Li.Et + Diazepam	25 mg/kg, p.o. +1 mg/kg, i.p.	1.267 ± 0.019 * (↑ 73%)	1.98 ± 0.034 * (↑ 88%)	1.43 ± 0.029 * (↑ 86%)
**X.**	Li.Et +Diazepam	50 mg/kg, p.o. +1 mg/kg, i.p.	1.03 ± 0.015 (↑ 71%)	1.58 ± 0.026 (↑ 85%)	1.26 ± 0.025 (↑ 83%)

Values are expressed as Mean ± SEM. *n* = 10; *** indicated *p* < 0.001, ** indicated *p* < 0.01 and * indicated *p* < 0.05 in comparison with diazepam. Parenthesis indicated % increase in SOD, CAT and GSH levels.

## Supplementary Materials

The following are available online at https://www.mdpi.com/1010-660X/55/5/192/s1. File S1: ARRIVE guidelines.

## References

[1] Mayeux R. (2010). Early Alzheimer’s disease. N. Eng. J. Med..

[2] Bachurin S.O., Bovina E.V., Ustyugov A.A. (2017). Drugs in clinical trials for Alzheimer’s disease: The major trends. Med. Res. Rev..

[3] Ovais M., Zia N., Ahmad I., Khalil A.T., Raza A., Ayaz M., Sadiq A., Ullah F., Shinwari Z.K. (2018). Phyto-therapeutic and nanomedicinal approach to cure Alzheimer disease: Present status and future opportunities. Front. Aging Neurosci..

[4] Kumar A., Singh A. (2015). A review on Alzheimer’s disease pathophysiology and its management: An update. Pharmacol. Rep..

[5] Rice-Evans C.A., Miller N.J., Paganga G. (1996). Structure-antioxidant activity relationships of flavonoids and phenolic acids. Free Radic. Biol. Med..

[6] Sadiq A., Zeb A., Ullah F., Ahmad S., Ayaz M., Rashid U., Muhammad N. (2018). Chemical characterization, analgesic, antioxidant, and anticholinesterase potentials of essential oils from *Isodon rugosus* Wall. ex. Benth. Front. Pharmacol..

[7] Ayaz M., Junaid M., Ullah F., Sadiq A., Khan M.A., Ahmad W., Shah M.R., Imran M., Ahmad S. (2015). Comparative chemical profiling, cholinesterase inhibitions and anti-radicals properties of essential oils from *Polygonum hydropiper* L.: A preliminary anti-Alzheimer’s study. Lipids Health Dis..

[8] Devasagayam T., Tilak J., Boloor K., Sane K.S., Ghaskadbi S.S., Lele R. (2004). Free radicals and antioxidants in human health: Current status and future prospects. J. Assoc. Physicians India.

[9] Summers W.K., Martin R.L., Cunningham M., DeBoynton V.L., Marsh G.M. (2010). Complex antioxidant blend improves memory in community-dwelling seniors. J. Alzheimer’s Dis..

[10] Frank B., Gupta S. (2005). A review of antioxidants and Alzheimer’s disease. Ann. Clin. Psychiatry.

[11] Danta C.C., Piplani P. (2014). The discovery and development of new potential antioxidant agents for the treatment of neurodegenerative diseases. Expert Opin. Drug Discov..

[12] De Kloet E.R., Joëls M., Holsboer F. (2005). Stress and the brain: From adaptation to disease. Nature Rev. Neurosci..

[13] Ovais M., Khalil A.T., Raza A., Islam N.U., Ayaz M., Saravanan M., Ali M., Ahmad I., Shahid M., Shinwari Z.K. (2018). Multifunctional theranostic applications of biocompatible green-synthesized colloidal nanoparticles. Appl. Microbial. Biotechnol..

[14] Yuen E.Y., Liu W., Karatsoreos I.N., Feng J., McEwen B.S., Yan Z. (2009). Acute stress enhances glutamatergic transmission in prefrontal cortex and facilitates working memory. Proc. Natl. Acad. Sci. USA.

[15] Esch T., Stefano G.B., Fricchione G.L., Benson H. (2002). The role of stress in neurodegenerative diseases and mental disorders. Neuro Endocrinol. Lett..

[16] Ayaz M., Junaid M., Ullah F., Subhan F., Sadiq A., Ali G., Ovais M., Shahid M., Ahmad A., Wadood A. (2017). Anti-Alzheimer’s studies on β-sitosterol isolated from *Polygonum hydropiper* L.. Front. Pharmacol..

[17] Linden D., Martinez J.L. (1986). Leu-enkephalin impairs memory of an appetitive maze response in mice. Behav. Neurosci..

[18] Ader R., de Wied D. (1972). Effects of lysine vasopressin on passive avoidance learning. Psychon. Sci..

[19] Lynch M. (2004). Long-term potentiation and memory. Physiol. Rev..

[20] Cragg G.M., Newman D.J., Snader K.M. (1997). Natural products in drug discovery and development. J. Nat. Prod..

[21] Atanasov A.G., Waltenberger B., Pferschy-Wenzig E.-M., Linder T., Wawrosch C., Uhrin P., Temml V., Wang L., Schwaiger S., Heiss E.H. (2015). Discovery and resupply of pharmacologically active plant-derived natural products: A review. Biotechnol. Adv..

[22] Ayaz M., Sadiq A., Junaid M., Ullah F., Subhan F., Ahmed J. (2017). Neuroprotective and anti-aging potentials of essential oils from aromatic and medicinal plants. Front. Aging Neurosci..

[23] Ovais M., Khalil A.T., Islam N.U., Ahmad I., Ayaz M., Saravanan M., Shinwari Z.K., Mukherjee S. (2018). Role of plant phytochemicals and microbial enzymes in biosynthesis of metallic nanoparticles. Appl. Microbial. Biotechnol..

[24] Ayaz M., Sadiq A., Wadood A., Junaid M., Ullah F., Khan N.Z. (2019). Cytotoxicity and molecular docking studies on phytosterols isolated from *Polygonum hydropiper* L.. Steroids.

[25] Ayaz M., Subhan F., Sadiq A., Ullah F., Ahmed J., Sewell R. (2017). Cellular efflux transporters and the potential role of natural products in combating efflux mediated drug resistance. Front. Biosci..

[26] Boubaya A., Hannachi H., Marzougui N., Triki T., Guasmi F., Ferchichi A. (2013). Genetic diversity assessment of *Lawsonia inermis* germplasm in Tunisian coastal oases by ISSR and RAPD markers. Dendrobiology.

[27] Bonjar S. (2004). Evaluation of antibacterial properties of some medicinal plants used in Iran. J. Ethnopharmacol..

[28] Al-Omar M.A. (2005). Dye of henna, on aldehyde oxidase activity in guinea pig liver. J. Med. Sci..

[29] Bhandarkar M., Khan A. (2003). Protective effect of *Lawsonia alba* lam., against CCI_4_ induced hepatic damage in albino rats. Indian J. Exp. Biol..

[30] Khan M.A.A., Jain D., Bhakuni R., Zaim M., Thakur R. (1991). Occurrence of some antiviral sterols in Artemisia annua. Plant Sci..

[31] Singh V., Pandey D. (1988). Fungitoxic studies on bark extract of *Lawsonia inermis* against ringworm fungi. Hindustan Antibiot. Bull..

[32] Shiksharthi A., Mittal S., Ramana J. (2011). Systematic review of herbals as potential memory enhancers. Int. J. Res. Pharm. Biomed. Sci..

[33] Aggarwal B.B., Sundaram C., Malani N., Ichikawa H. (2007). Curcumin: The Indian solid gold. The Molecular Targets and Therapeutic Uses of Curcumin in Health and Disease.

[34] Shah S.M., Ullah F., Ayaz M., Sadiq A., Hussain S., Shah A., Shah S.A.A., Ullah N., Ullah F., Ullah I. (2019). Benzoic acid derivatives of *Ifloga spicata* (Forssk.) Sch.Bip. as potential anti-Leishmanial against *Leishmania tropica*. Processes.

[35] Ayaz M., Junaid M., Ullah F., Sadiq A., Shahid M., Ahmad W., Ullah I., Ahmad A., Syed N.-i.-H. (2017). GC-MS analysis and gastroprotective evaluations of crude extracts, isolated saponins, and essential oil from *Polygonum hydropiper* L.. Front. Chem..

[36] Garber J., Barbee R., Bielitzki J., Clayton L., Hendriksen C., Donovan J. (2010). Guide for the Care and Use of Laboratory Animals.

[37] OECD (2001). Acute oral toxicity—Fixed dose procedure. OECD Guidelines for Testing of Chemicals.

[38] Vasudevan M., Parle M. (2007). Memory enhancing activity of Anwala Churna (*Emblica officinalis* Gaertn.): An ayurvedic preparation. Physiol. Behav..

[39] Parle M., Dhingra D., Kulkarni S. (2004). Improvement of mouse memory by *Myristica fragrans* seeds. J. Med. Food.

[40] Joshi H., Parle M. (2006). Zingiber officinale: Evaulation of its nootropic effect in mice. AJTCAM.

[41] Parle M., Dhingra D. (2003). Ascorbic acid: A promising memory-enhancer in mice. J. Pharmacol. Sci..

[42] Kumar M.V., Gupta Y. (2002). Effect of different extracts of *Centella asiatica* on cognition and markers of oxidative stress in rats. J. Ethnopharmacol..

[43] Anwar F., Hira S., Ahmad B., Saleem U. (2017). Antioxidants attenuate isolation- and L-DOPA-induced aggression in mice. Front. Pharmacol..

[44] Bhangale J.O., Acharya S.R. (2016). Anti-Parkinson activity of petroleum ether extract of *Ficus religiosa* (L.) leaves. Adv. Pharmacol. Sci..

[45] Kath R., Gupta R. (2006). Antioxidant activity of hydroalcoholic leaf extract of *Ocimum sanctum* in animal models of peptic ulcer. Indian J. Physiol. Pharmacol..

[46] Zohra T., Ovais M., Khalil A.T., Qasim M., Ayaz M., Shinwari Z.K. (2019). Extraction optimization, total phenolic, flavonoid contents, HPLC-DAD analysis and diverse pharmacological evaluations of *Dysphania ambrosioides* (L.) mosyakin & clemants. Nat. Prod. Res..

[47] Weissgerber T.L., Garcia-Valencia O., Garovic V.D., Milic N.M., Winham S.J. (2018). Meta-research: Why we need to report more than ’data were analyzed by t-tests or ANOVA’. eLife.

[48] Stein S., Mirokhin D., Tchekhovskoi D., Mallard G., Mikaia A., Zaikin V., Clifton C. (2002). The NIST Mass Spectral Search Program for the NIST/EPA/NIH Mass Spectra Library.

[49] Spencer J.P. (2008). Food for thought: The role of dietary flavonoids in enhancing human memory, learning and neuro-cognitive performance: Symposium on ‘diet and mental health’. Proc. Nutr. Soc..

[50] Longo F.M., Massa S.M. (2004). Neuroprotective strategies in Alzheimer’s disease. NeuroRx.

[51] Kouémou1 N.E., Taiwe G.S., Moto F.C.O., Pale S., Gwladys N.T., Njapdounke J.S.K., Nkantchoua G.C.N., Pahaye D., Ngo Bum E. (2017). Nootropic and neuroprotective effects of *Dichrocephala integrifolia* on scopolamine mouse model of Alzheimer’s disease. Front. Pharmacol..

[52] Olayinka O.O., Mbuyi N.N. (2014). Epidemiology of dementia among the elderly in sub-Saharan Africa. Int. J. Alzheimers Dis..

[53] Campos A.C., Fogaca M.V., Aguiar D.C., Guimaraes F.S. (2013). Animal models of anxiety disorders and stress. Braz. J. Psychiatry.

[54] El-Sherbiny D.A., Khalifa A.E., Attia A.S., Eldenshary E.E.-D.S. (2003). *Hypericum perforatum* extract demonstrates antioxidant properties against elevated rat brain oxidative status induced by amnestic dose of scopolamine. Pharmacol. Biochem. Behav..

[55] Gupta Y., Chugh A., Seth S. (1989). Opposing effect of apomorphine on antinociceptive activityfx of morphine: A dose-dependent phenomenon. Pain.

[56] Bhattacharya S., Satyan K.S., Ghosal S. (1997). Antioxidant activity of glycowithanolides from *Withania somnifera*. Indian J. Exp. Biol..

[57] Esposito M.D., Detre J.A., Alsop D.C., Shin R.K. (1995). The neural basis of the central executive system of working memory. Nature.

[58] Hayes J.D., McLellan L.I. (1999). Glutathione and glutathione-dependent enzymes represent a co-ordinately regulated defence against oxidative stress. Free Rad. Res..

[59] Scandalios J.G. (1993). Oxygen stress and superoxide dismutases. Plant Physiol..

[60] Pinchuk I., Shoval H., Dotan Y., Lichtenberg D. (2012). Evaluation of antioxidants: Scope, limitations and relevance of assays. Chem. Physics Lipids.

[61] Youdim K.A., Dobbie M.S., Kuhnle G., Proteggente A.R., Abbott N.J., Rice-Evans C. (2003). Interaction between flavonoids and the blood-brain barrier: In vitro studies. J. Neurochem..

[62] Giordano S., Darley-Usmar V., Zhang J. (2014). Autophagy as an essential cellular antioxidant pathway in neurodegenerative disease. Redox boil..

[63] Ali M., Muhammad S., Shah M.R., Khan A., Rashid U., Farooq U., Ullah F., Sadiq A., Ayaz M., Ali M. (2017). Neurologically potent molecules from Crataegus oxyacantha; isolation, anticholinesterase inhibition, and molecular docking. Front. pharmacol..

